# The gracilis tendon autograft is a safe choice for orthopedic reconstructive procedures: a consecutive case series studying the effects of tendon harvesting

**DOI:** 10.1186/s12891-019-2520-5

**Published:** 2019-03-30

**Authors:** Jonas S. Nordin, Ola Olsson, Karl Lunsjö

**Affiliations:** 1 Department of Orthopedics, Helsingborg Hospital, Helsingborg, Sweden; 2 Faculty of Medicine, Lund University, Lund, Sweden

**Keywords:** Gracilis autograft, Ligament reconstruction, Coracoclavicular reconstruction, Acromioclavicular dislocation, Hamstring graft

## Abstract

**Background:**

The gracilis tendon is commonly used as an autograft to reconstruct torn tendons or ligaments in many parts of the body. Little is known about the subjective and functional outcome after gracilis tendon harvest. The aim of this study was to evaluate the outcome of the donor leg in patients undergoing such surgery.

**Methods:**

Patients with chronic acromioclavicular joint dislocations undergoing coracoclavicular ligament reconstructions using autogenous gracilis tendon grafts were eligible for this study. The graft harvesting procedure was carried out in a standard fashion using a tendon stripper. Knee injury and Osteoarthritis Outcome Score (KOOS) were collected preoperatively and after 12 months. The first 5 patients were included retrospectively and lacked preoperative data, for these patients age- and gender matched normative KOOS scores were used as baseline values. Isometric knee flexor strength in 60° and 90° degrees of flexion was measured at final follow up at a median of 26 (14–56) months postoperatively with the non-operated leg used as reference.

**Results:**

Twenty four patients were eligible for the study and 2 were excluded. The 22 patients available for analysis had a mean age of 44 (22–62) years at the time of surgery and 4 were women. There was no statistically significant change in KOOS 12 months postoperatively compared to baseline values but the patients were weaker in knee flexion in the operated leg compared to the non-operated one.

**Conclusions:**

Gracilis tendon harvesting results in a weakness of knee flexion but does not impair subjective knee function and is a procedure that can be recommended when an autogenous tendon graft is needed.

## Background

The gracilis tendon is often used as an autograft for ligament or tendon reconstruction. Most commonly it is used in combination with the semitendinosus tendon as a hamstring graft in anterior cruciate ligament (ACL) reconstructions, but it is also frequently used in other knee reconstructive procedures as well as in shoulder, elbow, hip and ankle surgery [1–6]. While allografts are an alternative to autografts there are indications that they stretch more postoperatively and they are not readily available in all countries [7, 8]. In 2011, at our institution, we started using gracilis autografts in coracoclavicular (CC) ligament reconstructions for treatment of chronic acromioclavicular joint dislocations [9].

While graft site morbidity and knee flexor strength after ACL reconstruction using hamstring grafts has been thoroughly studied [10, 11], there is little evidence regarding the knee related outcome after isolated gracilis tendon harvesting from a limb in which no reconstructive procedure is planned. Two studies touching on this subject concern patients undergoing primary, unilateral ACL reconstructions that were randomized with regard to which leg the hamstring graft would be harvested from [12, 13]. Both of these trials report that graft harvesting is associated with a decrease in knee flexor strength that is resolved within 12 months postoperatively and they do not advice against using the unaffected leg as a graft donor site.

While providing important information, the trials above studied the effect of harvesting a two-tendon hamstring autograft for use in ACL-reconstruction and these results cannot be directly translated to a situation where an isolated gracilis graft is used for reconstructive procedures in other parts of the body than the knee. After reviewing the literature we could find only one study investigating this issue in which 22 patients were retrospectively included after having undergone gracilis tendon harvesting to provide grafts for various foot and ankle procedures [1]. The patients were significantly weaker in knee flexion in the donor leg compared to the contralateral one but displayed minimal donor site morbidity.

In our study we aim to present further evidence regarding the outcome and possible morbidity related to gracilis tendon harvesting. This will allow surgeons to perform a more detailed risk-benefit analysis and patients to receive a more accurate preoperative information. Our hypothesis is that gracilis tendon harvesting leads to a weakening in knee flexion but not a reduction in subjective knee function.

## Materials and methods

This study comprised 24 consecutive patients operated between 2011 and 2016 with CC ligament reconstruction using an autogenous gracilis tendon graft. The hypothesis for this study was formulated in the end of 2011 after 5 patients had already undergone surgery, only postoperative outcome measures were available for these 5 patients and the study is therefore of combined prospective and retrospective design.

### Participants

Patients between 18 and 75 years of age with chronic, symptomatic acromioclavicular joint dislocations that were planned for CC ligament reconstruction using autogenous gracilis tendon grafts were eligible for the study. Patients were excluded if they had unresolved knee injuries, ongoing joint diseases that affected the knee, previous knee surgery, other conditions that were likely to greatly effect outcome or were unable to understand or comply with postoperative rehabilitation protocols because of mental or systemic disease. The 5 patients operated before November 2011 were retrospectively included and preoperative outcome measures are therefore missing, the remaining 19 patients were prospectively included in the study.

### Surgical technique

The patients underwent CC ligament reconstruction using two different methods. The first 8 patients were operated using the GraftRope® device (Arthrex Inc., Naples, FL) and for the remaining patients the anatomic coracoclavicular reconstruction technique was used [14]. The surgical technique was changed because of shoulder related complications with the GraftRope® device [9]. To reconstruct the coracoclavicular ligaments an autogenous gracilis tendon graft was used in all cases, the surgical technique to harvest the graft was identical regardless of which reconstructive procedure was performed in the shoulder.

The tendon grafts were harvested immediately before the shoulder surgery and both the knee and shoulder were prepped and draped. Under general anesthesia and in a bloodless field a 4 cm incision was made over the pes anserinus, the saphenous nerve and infrapatellar branches were protected if located and the sartorius fascia incised. The common insertion of the semitendinosus and gracilis tendon was located and the gracilis tendon was identified as the most anterior of the two. Adhesions were bluntly dissected and the tendon was then harvested using a tendon stripper. The sartorius fascia and subcutaneous tissue was closed using absorbable sutures and the skin was closed using non-absorbable sutures. After a soft dressing was applied the tourniquet was deflated and the shoulder surgery was begun.

Full mobilization of the leg was allowed postoperatively but patients were given a crutch to allow for partial load bearing if pain required it. All patients were advised to perform physiotherapy but no supervised training was ordered as part of this study.

### Outcome measures

The primary outcome measure was the Knee injury and Osteoarthritis Outcome Score (KOOS). The questionnaire was completed 12 months postoperatively by all patients and scores were compared to preoperative ones for the prospectively included participants. For the patients included retrospectively preoperative scores were not available, therefore age- and gender matched normative values from a study of the general population were used for statistical analysis [15].

Isometric knee flexor strength was the secondary outcome measure. Testing was performed during a follow up visit for all included patients at the end of the study period. An IsoForceControl® EVO2 dynamometer (MDS Medical Devise Solutions AG, Oberburg, Switzerland) was placed on a vertical surface perpendicular to the direction of the force. The patient was in a prone position on an examination table with the strap from the dynamometer around the ankle. Instructions were given to hold on to the edges of the table and keep the pelvis low during attempts, no stabilization of the pelvis was used. After a trial attempt using submaximal force testing was performed on both the operated and non-operated leg with the knee in both 60° and 90°, three attempts per leg and angle were completed. In half of the patients the operated leg was tested first and in the other half the non-operated. The mean of the two highest values for each leg and angle was used for statistical analysis.

To find patients with clinically severe donor site related complications, such as infection, hematoma or muscle rupture, a medical journal review of the hospitals’ database was performed at the end of the study period.

### Statistics

For KOOS scores each subscale was analyzed separately and change from baseline at 12 months was analyzed using the Wilcoxon signed-rank test. For the participants that did not have preoperative scores, age- and gender matched normative values were used.

For the isometric knee flexor strength all analyses are performed as paired calculations. The relative force of the operated leg was calculated by dividing the values of this leg by those of the non-operated one. The actual difference between the legs was calculated by subtracting the force of the non-operated from that of the operated one. These calculations were performed for each patient separately and these values were then used for further calculations. The Wilcoxon signed-rank test was used to analyze if the actual difference and relative force differed from 0 and 1 respectively. Statistical significance was set at *p* < 0.05. All analyses were performed using SAS 9.4 (SAS Institute Inc., Cary, NC, USA).

## Results

A total of 24 patients were eligible for the study and two were excluded. One due to mental illness and one because of chronic pain syndrome, both of these patients were from the group included prospectively. Out of the 22 patients available for follow up there were 4 females, the mean age was 44 years (22–62) and the graft was harvested from the right leg in 14 cases.

**Fig. 1:**
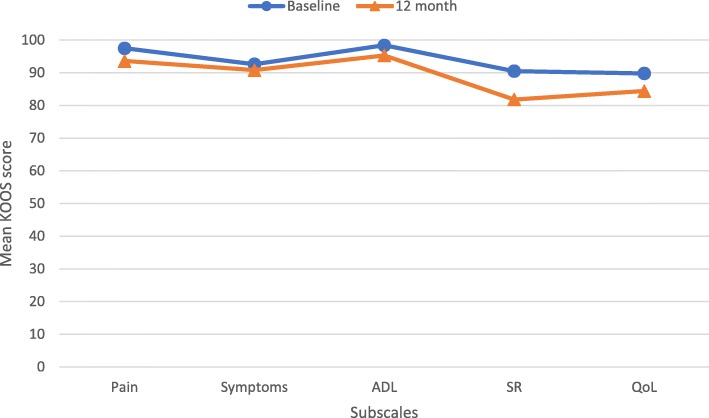
KOOS profile presenting mean KOOS scores for each subscale. ADL, activities of daily living; SR, sports and recreation; QoL, quality of life

The five KOOS subscales, pain, symptoms, activities of daily living (ADL), sports and recreation (SR) and quality of life (QoL), were analyzed separately and the mean scores are presented in Fig. 1. There was no statistically significant change between baseline and 12 month values for any of the KOOS subscales.

Testing of isometric hamstring strength was performed at a median of 26,5 months (14–56) postoperatively and was completed by all of the 22 patients. The operated leg was significantly weaker in both 60° and 90° of flexion performing a mean force of 93 and 83% of the non-operated leg in the two angles respectively. Complete results are shown in Table 1.

**Table 1:** Isometric knee flexion strength

Angle		Operated leg (N)	Non-operated leg (N)	Actual difference (N)	Relative difference
60°	Mean Force (SD)	199.7 (78.2)	212.4 (76.1)	−12.7 (21.6)	0.93 (0.1)
	*P*-value			0.0051	0.0025
90°	Mean Force (SD)	123.1 (54.6)	145.9 (54.7)	−22.7 (27.0)	0.83 (0.19)
	*P*-value			0.0002	0.0002

Mean force is presented in Newtons. Actual difference is calculated by subtracting the force of the non-operated leg from that of the operated leg. Relative difference is calculated by dividing the force of the operated leg by that of the non-operated leg. *SD* standard deviation. *p*-values from the paired Wilcoxon signed-rank test

Analysis of the hospitals’ medical journal database revealed that one patient had problems with a slow healing incision with no signs of infection and the same patient suffered a minor hamstring sprain after stumbling. Other than these two episodes that both resolved spontaneously no donor site complications were found.

## Discussion

This study shows that there was no statistically significant decrease in KOOS scores in any of the subscales 12 months after gracilis tendon harvesting compared to baseline values. This indicates that the use of autogenous gracilis grafts is well tolerated by patients and does not impair subjective knee function. However, the donor limb was significantly weaker in isometric knee flexion compared to the unoperated side.

While the outcome after ACL reconstruction using ipsilateral hamstring grafts has been thoroughly studied there are very few articles available on the effects of isolated gracilis tendon grafting from a knee not planned for reconstructive surgery. To our knowledge the study by Cody et al. is the only one investigating this issue [1]. In their retrospective study 70 patients had undergone hamstring tendon autografting for use in foot and ankle reconstructive procedures and in 22 of these an isolated gracilis tendon graft had been harvested. The patients were interviewed postoperatively and 95% of the patients responded that they were either satisfied or very satisfied with their operative result and all patients would recommend the surgery to someone else. There were no serious donor site sequelae and the authors conclude that hamstring autografts can be used with high patient satisfaction and minimal morbidity. The study by Cody et al. is limited by a follow up rate of only just above 50% and by the fact that they did not use a patient related outcome measure for follow up. In our study we used the validated outcome measure KOOS that provides a more comprehensive evaluation of the knee and leg function and we can confirm the findings by Cody et al. [1].

Cody et al. also measured isokinetic knee flexion strength between 13 and 56 months postoperatively [1]. They found that gracilis tendon harvest lead to a significant decrease in strength compared to the unoperated side. In our study we measured isometric and not isokinetic force but as the two are very closely related [16] our results are comparable. In accordance with Cody et al. [1] we found that the donor leg strength was 93% of the non-operated side when tested at 60° of knee flexion and 83% at 90°. Considering that both Cody et al. [1] and this study showed that the subjective outcome of the donor leg was not affected by the surgery the measurable decrease in strength seems to be clinically unimportant in our study populations. It is, however, possible that the hamstring strength deficit might be of clinical importance in patients with very high demands such as those participating in certain sports. It has been suggested that a weakness in knee flexion might increase the risk of ACL tears [17] and it has also been shown that low hamstring strength is a risk factor for hamstring strain injuries in professional football players [18]. Structured physiotherapy has been shown effective in rebuilding hamstring strength [12] and should, therefore, be considered after gracilis tendon harvest.

There are two further studies concerning the outcome after hamstring tendon harvesting without further knee related procedures in the donor limb. In the study by Yasuda et al. 70 patients were randomized to have a two-tendon hamstring graft harvested from either the ipsilateral or contralateral leg when undergoing planned ACL-reconstructions, 34 patients were included in the contralateral group [12]. All subjective problems, such as activity related soreness, restricted range of motion, and reduction in the points in the Cincinnati sports medicine center rating scale, were resolved by 3 months postoperatively. In the second, similar, study by McRae et al., 50 patients were randomized to the contralateral harvest group [13]. The main outcome variable was the ACL quality of life outcome measure and there was no difference between groups at any point in time during follow up. The score did increase with time at each of the three follow up visits until 12 months postoperatively but no further improvement was seen at 24 months. While our study cannot be directly compared to the two above because of the differences in tendons harvested and follow up protocols we are able to provide further evidence that the negative effects of hamstring tendon grafting are resolved by one year postoperatively.

A strength of our study is that we can confirm the previous findings of Cody et al. [1] that isolated gracilis tendon harvesting leads to a reduction of knee flexion strength. We also deepen the understanding of the subjective outcome after this procedure by being the only study presenting the results of a validated patient related outcome measure. As gracilis autografts are widely used in orthopedic procedures outside of knee surgery these results are important to establish the safety and outcome of the harvesting procedure in such settings and allow surgeons to provide patients a detailed preoperative information [1–6].

The main weakness of this study is that 5 of the 22 patients underwent surgery before the study was started and are therefore lacking preoperative outcome measures. In these 5 cases we compared postoperative KOOS scores to age and gender matched normative values acquired from a population based study of 568 persons [15]. Further, while there was no statistically significant decrease in KOOS scores one year postoperatively we did see a slight reduction in each of the five subscales and with a larger sample size this might have turned out to be significant. The differences were, however, not larger than what is considered the minimal clinically important change in any of the subscales [19]. Another weakness is that we did not systematically examine the donor site in the post operative period to look for local complications such as wound infection, hematoma or muscle rupture. Instead we used medical journal review in the Hospitals’ database to register complications that the patients sought care for. It is possible that patients with complications have been treated at other caregivers but this is unlikely as we are the only orthopedic clinic in the area and postoperative complications are usually referred to us. Further, we only measured isometric knee flexion strength and did not assess isokinetic performance, but as the two are closely related and our results are in accordance with other studies we do not believe this choice of method to have caused erroneous conclusions [1, 16].

## Conclusion

Gracilis tendon harvesting results in a weakness of knee flexion but does not have significant impact on subjective knee function and is a procedure that can be recommended when an autogenous tendon graft is needed.

## Abbreviations

ACL: Anterior cruciate ligament
ADL: Activities of daily living
CC: Coracoclavicular
KOOS: Knee injury and Osteoarthritis Outcome Score
QoL: Quality of life
SR: Sports and recreation
